# Cannabinoids Reduce Inflammation but Inhibit Lymphocyte Recovery in Murine Models of Bone Marrow Transplantation

**DOI:** 10.3390/ijms20030668

**Published:** 2019-02-04

**Authors:** Iman Khuja, Zhanna Yekhtin, Reuven Or, Osnat Almogi-Hazan

**Affiliations:** Laboratory of Immunotherapy and Bone Marrow Transplantation, Hadassah Medical Center, The Faculty of Medicine, Hebrew University of Jerusalem, 91120 Jerusalem, Israel; iman.khuja@mail.huji.ac.il (I.K.); zhannay@hadassah.org.il (Z.Y.); reuvenor@hadassah.org.il (R.O.)

**Keywords:** hematopoiesis, bone marrow transplantation, graft versus host disease, lymphocyte, immune, cannabis, phytocannabinoids, D9 tetrahydrocannabinol, cannabidiol, cannabinoid receptor 2

## Abstract

Cannabinoids, the biologically active constituents of Cannabis, have potent neuronal and immunological effects. However, the basic and medical research dedicated to medical cannabis and cannabinoids is limited. The influence of these treatments on hematologic reconstitution and on the development of graft versus host disease (GVHD) after bone marrow transplantation (BMT) is largely unknown. In this research, we compared the influence of D9 tetrahydrocannabinol (THC) and cannabidiol (CBD) on lymphocyte activation in vitro and in murine BMT models. Our in vitro results demonstrate that these treatments decrease activated lymphocyte proliferation and affect cytokine secretion. We also discovered that CBD and THC utilize different receptors to mediate these effects. In vivo, in a syngeneic transplantation model, we demonstrate that all treatments inhibit lymphocyte reconstitution and show the inhibitory role of the cannabinoid receptor type 2 (CB2) on lymphocyte recovery. Although pure cannabinoids exhibited a superior effect in vitro, in an allogeneic (C57BL/6 to BALB/c) BMT mouse model, THC-high and CBD-high cannabis extracts treatment reduced the severity of GVHD and improved survival significantly better than the pure cannabinoids. Our results highlights the complexity of using cannabinoids-based treatments and the need for additional comparative scientific results.

## 1. Introduction

In recent years, there has been a rapid increase in the medical use of cannabis (Marijuana). While cannabis is not registered as a drug or a medical product, the potential of cannabis-based medicines for the treatment of various conditions [1] has led many countries around the world to authorize the clinical use of such treatments. However, the basic and medical research dedicated to medical cannabis is currently limited.

Cannabis contains numerous molecules, including more than 60 chemical compounds classified as cannabinoids, and the different sub-strains vary in their cannabinoid composition [2]. Two cannabinoids have been the focus of most of the studies examining medical uses: D9 tetrahydrocannabinol (THC) and cannabidiol (CBD). THC and some of the other cannabinoids mediate their actions primarily through the Gi protein-coupled seven transmembrane cannabinoid receptors: (1) Cannabinoid receptor 1 (CB1), which is mainly expressed in the brain and to some extent in peripheral tissues such as immune tissues and (2) Cannabinoid receptor type 2 (CB2), which is highly expressed in immune cells. The expression of CB2 is higher in lymph nodes and spleen than in peripheral blood cells and is different in various immune cell populations (B cells > NK cells > monocytes > neutrophils > CD8 T cells > CD4 T cells) [3]. CBD has a very weak affinity to CB1 and CB2 [4]. Several reports have demonstrated CBD signaling through non-cannabinoid receptor mechanisms, such as transient receptor potential cation (TRP) channels, G protein-coupled receptor 55 (GPR55) and the nuclear receptor: Peroxisome Proliferator-Activated Receptor gamma (PPAR-γ) [5,6].

In addition to their effect on the nervous system, both phyto and endogenous cannabinoids have important immunological effects. They possess a wide range of anti-inflammatory properties as they induce the production of anti-inflammatory cytokines such as IL-4, IL-5 and IL-10, and affect the differentiation and function of several types of immune cells [7,8]. The involvement of cannabinoid receptor signaling in the biology of hematopoietic stem and progenitor cells has also been reported [9,10]. Importantly, different cannabinoids were shown to differentially affect immune cell function [11].

Bone Marrow Transplantation (BMT) is a well-established treatment for malignant and non-malignant hematological diseases [12]. Allogeneic BMT can cause the inflammatory condition, Graft versus Host Disease (GVHD), a major cause of morbidity and mortality in BMT patients [13]. In addition, slow, impaired or dysregulated reconstitution of donor-derived immune cell populations, together with GVHD and other post-transplant complications, causes susceptibility to both common and rare infections. The early post-engraftment period is characterized by a progressive recovery of cell-mediated immunity; however, full reconstitution of the hematological components may take years [14].

Although there is a lot of information regarding the influence of cannabis and cannabinoids on the immune system, the effect of THC, CBD and cannabis extracts was never compared. In addition, the effect of these treatments on the reconstitution of the hematological system after BMT and their efficacy in treating GVHD patients is largely unknown. Moreover, the role of the endocannabinoid receptor CB2 in these processes is not clear. Studies in the past generally focused on a single cannabinoid. THC treatment was shown to reduce GVHD in a mouse semi-allogeneic model that did not include both conditioning regimen and BMT (C57BL/6 spleen cells into C57BL/6 × DBA/2 F1) [15], and a recent publication demonstrated the beneficial effect of the cannabinoid CBD in GVHD prophylaxis in patients [16], but the differential effects of the cannabinoids was not examined.

We hypothesize that each cannabinoid has selective effects on hematopoietic and immune cell differentiation and function, and hence, a different impact on hematopoiesis and GVHD. In our research, we compared the consequences of treatment with THC and CBD in vitro and in murine BMT models. Since it has been suggested that the combination of cannabinoids with other active molecules in the plant may achieve better clinical results than pure cannabinoids (known as the entourage effect) [17], we also examined the differences between the effects of the pure cannabinoids and high THC/high CBD cannabis extracts. We show here that all the treatments reduce activated lymphocyte proliferation in vitro, but pure cannabinoids, particularly CBD, have a stronger inhibitory effect. We also found that CBD and THC utilize different receptors to mediate these effects. Using a syngeneic transplantation model, we demonstrate that all treatments, pure THC in particular, inhibit lymphocyte reconstitution after transplantation; in addition, we also show the inhibitory role of the cannabinoid receptor CB2 on lymphocyte recovery. Although pure cannabinoids had a superior effect in vitro, cannabis extracts were better at reducing the severity of disease and improving survival in the GVHD model than pure cannabinoids.

Our results highlight both similarities and the differences between various cannabis-based drugs in BMT. As different strains of cannabis contain a wide range of cannabinoids and other molecules that may influence the clinical outcome of the treatment, a better understanding of the effects of each molecule on hematological recovery and GVHD pathology will assist physicians in providing the best possible treatment for their patients.

## 2. Results

### 2.1. CBD Is a Stronger Inhibitor of In Vitro Activated Lymphocyte Proliferation Than THC

In order to learn about the effects of pure CBD/THC and cannabis extracts on lymphocyte function, we decided to utilize in vitro methods first. Cannabis extracts with a high content (20–30%) of CBD or THC were named CBD Botanical Drug Substance (BDS) or THC BDS, respectively. We used these extracts in addition to the pure cannabinoids for two reasons: First, most patients are currently treated with cannabis-based medications and not with pure cannabinoids. Second, we wanted to examine the possible advantage of the entourage effect [17].

**Figure 1:**
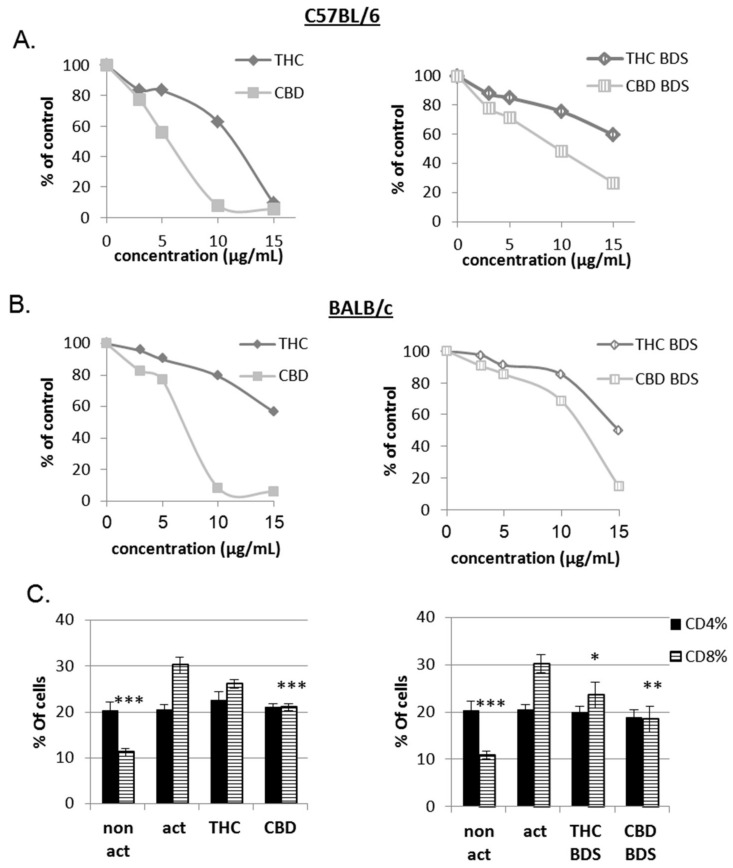

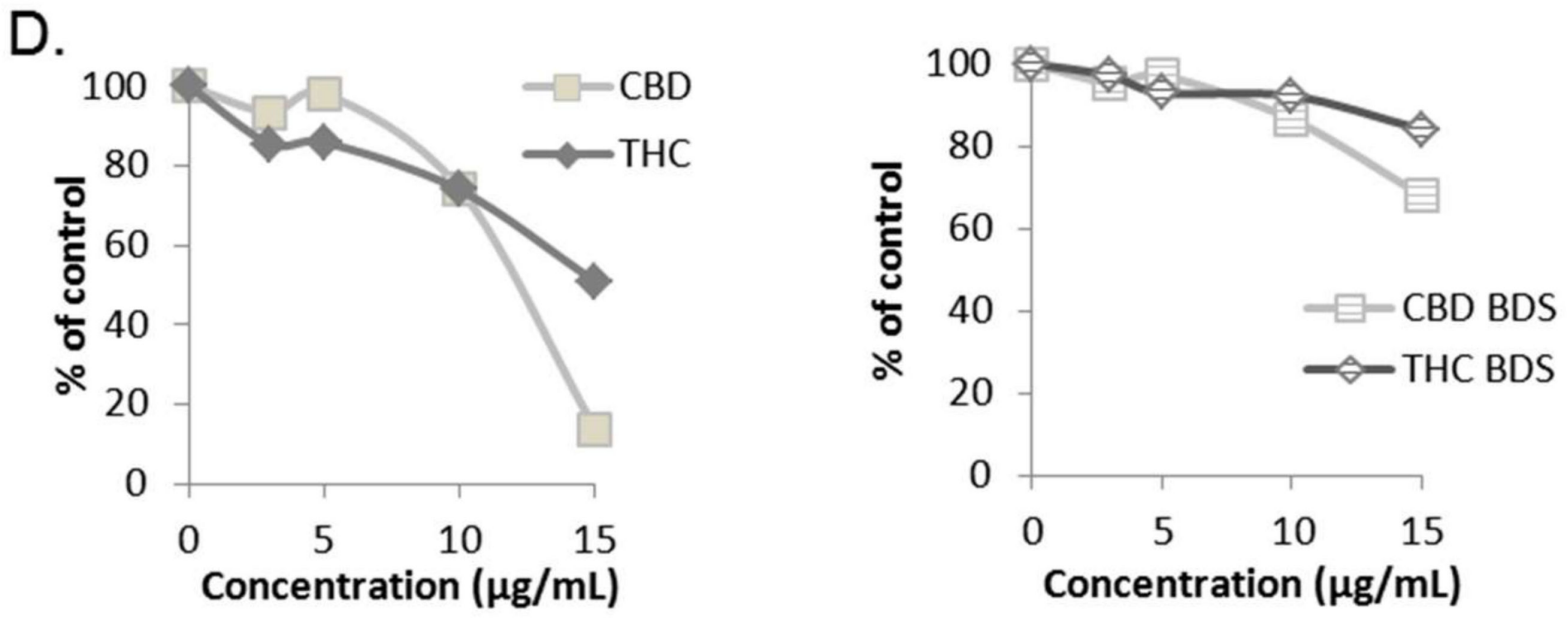
The influence of pure CBD/THC and cannabis extracts on lymphocyte activation. Proliferation of CFSE-stained, CD3-activated splenocytes from C57BL/6 (**A**,**C**) and Balb/c (**B**) mice was analyzed on day 4 after activation using flow cytometry analysis. (**A**) Summary of six independent experiments. When Comparing all treatments to control, the differences are significant starting from 3 µg/mL. The differences between THC/CBD and THC BDS/CBD BDS are significant starting from 5 µg/mL. (**B**) Summary of three independent experiments. When Comparing all treatments to control, THC to CBD and THC BDS to CBD BDS, significant differences were observed starting from 5 µg/mL. (**C**) Flow cytometry analysis of activated C57BL/6 splenocytes stained with anti-CD4 and anti-CD8 antibodies. Data is summarized from six independent experiments for pure cannabinoids and four independent experiments for BDS. Results are expressed as mean + SEM. *p* Value as compared to the activated control cells *, <0.05; **, <0.001; ***, <0.0001. (**D**) C57BL/6 splenocytes were activated for 4 h, stained with anti-CD69 antibodies and analyzed using flow cytometry. Data is summarized from three independent experiments. The differences of all treatments as compared to control are significant at 15 µg/mL, act: activated splenocytes, THC: D9 tetrahydrocannabinol, CBD: cannabidiol, BDS: Botanical Drug Substance.

The effect of cannabis/cannabinoids on the proliferation of activated lymphocytes was analyzed. Succinimidyl ester (CFSE)-labeled C57BL/6 or BALB/c mouse splenocytes were activated with anti-CD3 antibodies for 4 days in the presence of pure cannabinoids, CBD BDS or THC BDS at various concentrations. Cell proliferation was assessed using CFSE FACS analysis. Interestingly, in vitro the inhibitory effect of pure cannabinoids on lymphocyte activation was stronger than that of cannabis extracts. Whether in the form of pure cannabinoids or cannabis extract, CBD inhibited proliferation significantly better than THC (Figure 1A,B and Figure S1A). The treatments, in the concentrations used, were not toxic to the cells (Figure S1B). Similar results were obtained using human Peripheral Blood Mononuclear Cells (PBMC) (Figure S1C). Upon anti-CD3 activation, the percentage of CD8 cells in the samples is elevated (Figure 1C). CBD, CBD BDS and, to a lesser extent, THC BDS treatments significantly inhibited this elevation.

CD69 is a classical early marker of lymphocyte activation due to its rapid appearance on the surface of the plasma membrane after stimulation [18]. To test the effect of pure CBD/THC and cannabis extracts on CD69 cell surface expression, C57BL/6 mouse splenocytes were activated with anti-CD3 antibodies for 4 h in the presence of cannabinoid treatments. Cells were stained with anti-CD69 antibodies and expression was assessed using FACS analysis. The lower concentrations of all treatments had a non-significant effect on CD69 expression. Higher concentrations of 10–15 µg/mL induced inhibition of CD69 surface expression upon activation. CBD treatment had no effect in 3–5 µg/mL, but caused 87% inhibition in 15 µg/mL samples. In 15 µg/mL CBD BDS samples, surface expression of CD69 was reduced only by 22% (Figure 1D and Figure S2A).

Next, we used the supernatant from the C57BL/6 experiments (Figure 1A) to test the effect of cannabinoid treatment on cytokine secretion upon lymphocyte activation. We tested four different cytokines: IL-17, secreted in the Th17 response; IL-10 an indicator for immune regulation, secreted by Tregs and other cells; TNFα, secreted in the Th1 response; and IL-5, secreted in the Th2 response. The levels of secreted cytokines were examined using ELISA. We show the results obtained using 3 µg/mL treatment with pure cannabinoids and 10 µg/mL treatment with the cannabis extracts, which contain approximately 30% of the designated cannabinoid. The results for IL-17 and IL-10 after treatment with various other concentrations can be found in the Supplementary Data.

**Figure 2:**
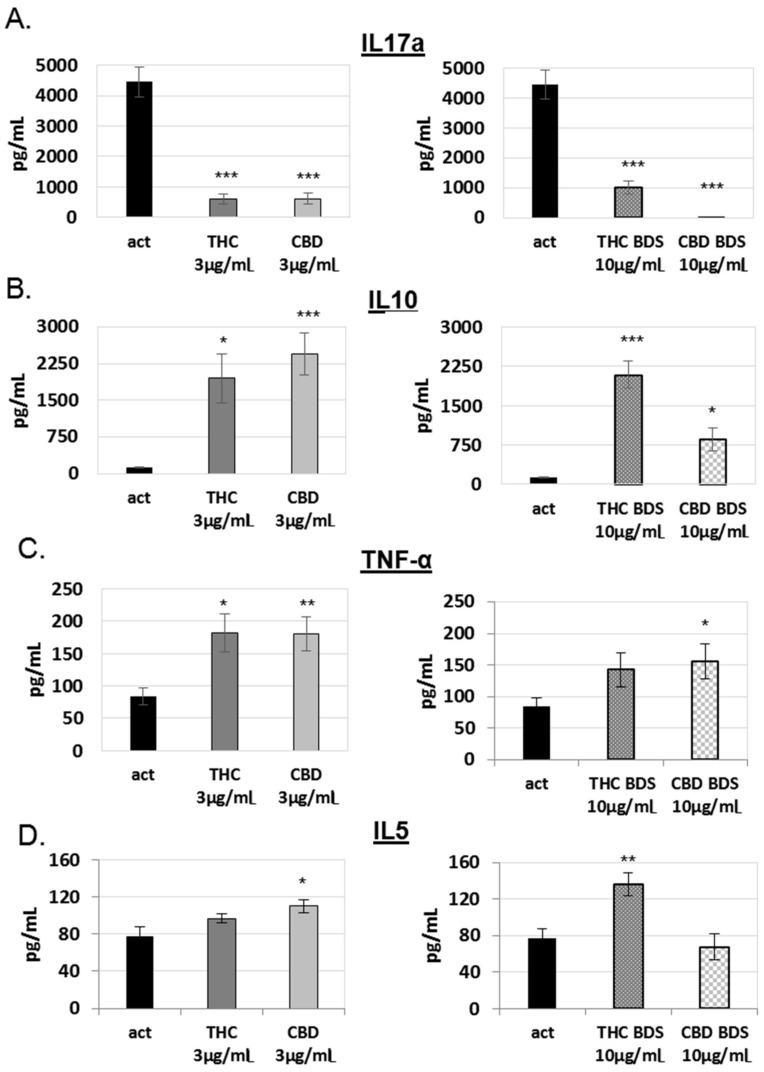
The influence of pure CBD/THC and cannabis extracts on cytokine secretion. Quantification of IL-17a (**A**), IL-10 (**B**), TNFα (**C**), and IL-5 (**D**) secretion from C57bl/6 splenocytes activated for 4 days which were treated with cannabinoids/cannabis, was performed using enzyme-linked immunosorbent assay on culture medium of activated cells. Data are summarized for five independent experiments for CBD BDS and six independent experiments for the other treatments. Results are expressed as mean + SEM. *p* Value as compared to activated control cells *, <0.05; **, <0.001; ***, <0.0001, act: activated splenocytes, THC: D9 tetrahydrocannabinol, CBD: cannabidiol, BDS: Botanical Drug Substance.

All treatments significantly reduced IL-17 secretion (Figure 2A, Figure S2). CBD BDS had the strongest effect with only 0.25% IL-17 in the supernatant as compared to untreated activated lymphocytes (control). IL-10 secretion was significantly increased by all treatments (Figure 2B, Figure S2). Pure CBD had the strongest effect, with 1806% IL-10 in the supernatant (compared to control). All treatments led to a small increase in TNFα secretion (Figure 2C), which was significant in all treatments except THC BDS. The levels of IL-5 secretion were affected only by THC BDS and pure CBD treatments (Figure 2D).

Overall, these results show that the cannabinoids CBD and THC have an inhibitory effect on lymphocyte activation, which is associated with a reduction in the secretion of the inflammatory IL-17 cytokine and an elevation in the secretion of the regulatory cytokine IL-10.

### 2.2. THC and CBD Utilize Different Receptors to Affect Lymphocyte Proliferation

**Figure 3:**
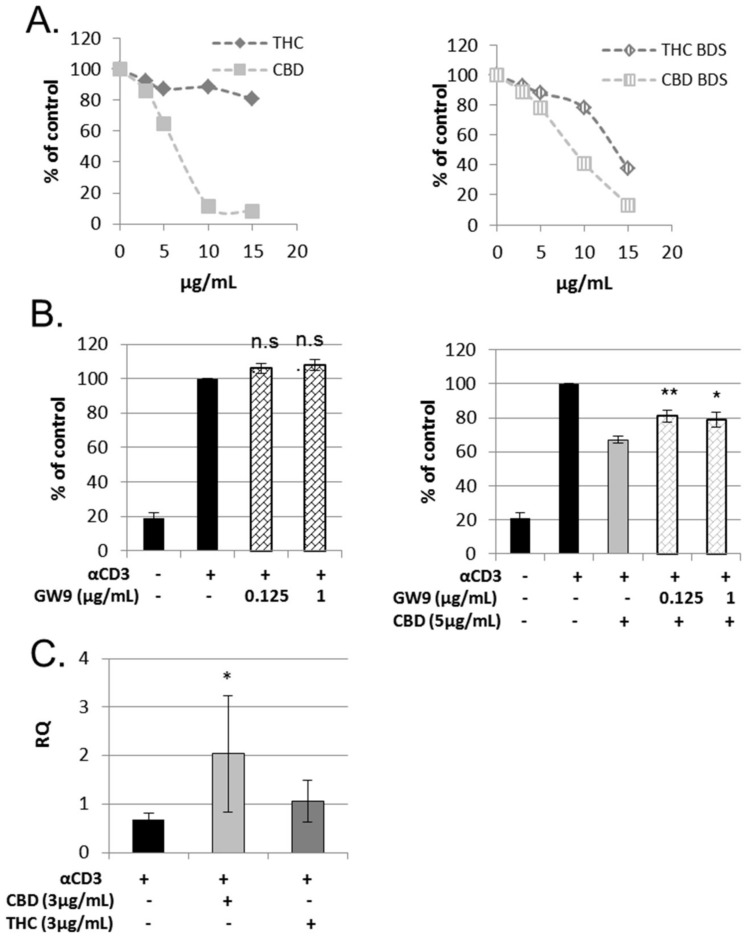
Receptors involved in THC and CBD’s effect on lymphocyte proliferation. (**A**) Proliferation of CFSE-stained, 4 days CD3-activated, splenocytes from CB2 knockout mice was analyzed using flow cytometry. Summary of four independent experiments. The differences of CBD, THC BDS and CBD BDS as compared to control are significant starting from 3 µg/mL. The differences of THC as compared to control are significant starting from 10 µg/mL. The differences of THC when compared to CBD is significant starting from 3 µg/mL. (**B**) The influence of PPARγ antagonist, GW9662, on CBD’s effect on lymphocyte activation. Proliferation of CFSE-stained, CD3-activated murine splenocytes was analyzed using flow cytometry analysis. Summary of eight independent experiments. *p* Value—samples were compared to act spl + CBD (right) or act spl (left). (**C**) Real time PCR analysis for the expression of cyp1a1 in activated splenocytes treated with THC or CBD. Summary of four independent experiments. Results are expressed as mean + SEM. *p* Value *, <0.05; **, <0.001;, act spl: activated splenocytes, THC: D9 tetrahydrocannabinol, CBD: cannabidiol, BDS: Botanical Drug Substance.

The cannabinoid receptor CB2 is highly expressed in immune cells [19,20]. To elucidate whether CB2 is involved in the effects of THC and CBD on lymphocytes, we used CB2 knock-out mice (CNR2^−/−^). First, we used splenocytes extracted from CNR2^−/−^ mice (Figure S3A,B) in a CFSE lymphocyte proliferation assay. The inhibitory effect of pure THC, but not pure CBD, was abolished in CNR2^−/−^-derived splenocytes (Figure 3A). Interestingly, the inhibitory effect of THC BDS was maintained.

Our results indicate that the CB2 receptor is the main mediator for THC’s effect on lymphocytes, whereas CBD’s effect clearly does not involve CB2 signaling. Several molecules have been proposed as mediators for CBD’s effects on mammalian cells [5,6]. To search for the molecules which are involved in CBD’s effect on lymphocyte activation, we used several inhibitors together with CBD in a CFSE lymphocyte proliferation assay. A967079, BCTC and GSK2193874 are antagonists to TRP channels TRPA1, TRPV1 and TRPV4 respectively, which have been demonstrated to mediate CBD signaling. However, we found that none of these antagonists interfered with CBD’s inhibitory effect on lymphocyte activation (Figure S4A–C). CID16020046, an antagonist to GPR55, also had no effect (Figure S4D). Another potential mediator of CBD signaling is the nuclear receptor PPAR-γ [21]. We found that GW9662, a PPAR-γ antagonist, could partially reverse the effect of CBD on lymphocyte proliferation (Figure 3B). The nuclear receptor aryl hydrocarbon receptor (AhR) is involved in the regulation of Treg and Th17 cell differentiation [22], and can be activated by cannabinoids, as demonstrated in a human hepatoma cell line [23]. Since CBD and THC affected IL-17 and IL-10 secretion from activated lymphocytes, we decided to test the possible function of these cannabinoids in AhR activation by examining the levels of expression of an AhR regulated gene, cyp1a1, in treated cells. Only CBD treatment significantly elevated the expression of cyp1a1 (Figure 3C).

### 2.3. Cannabinoid Treatment Alters Hematologic Recovery after Bone Marrow Transplantation

**Figure 4:**
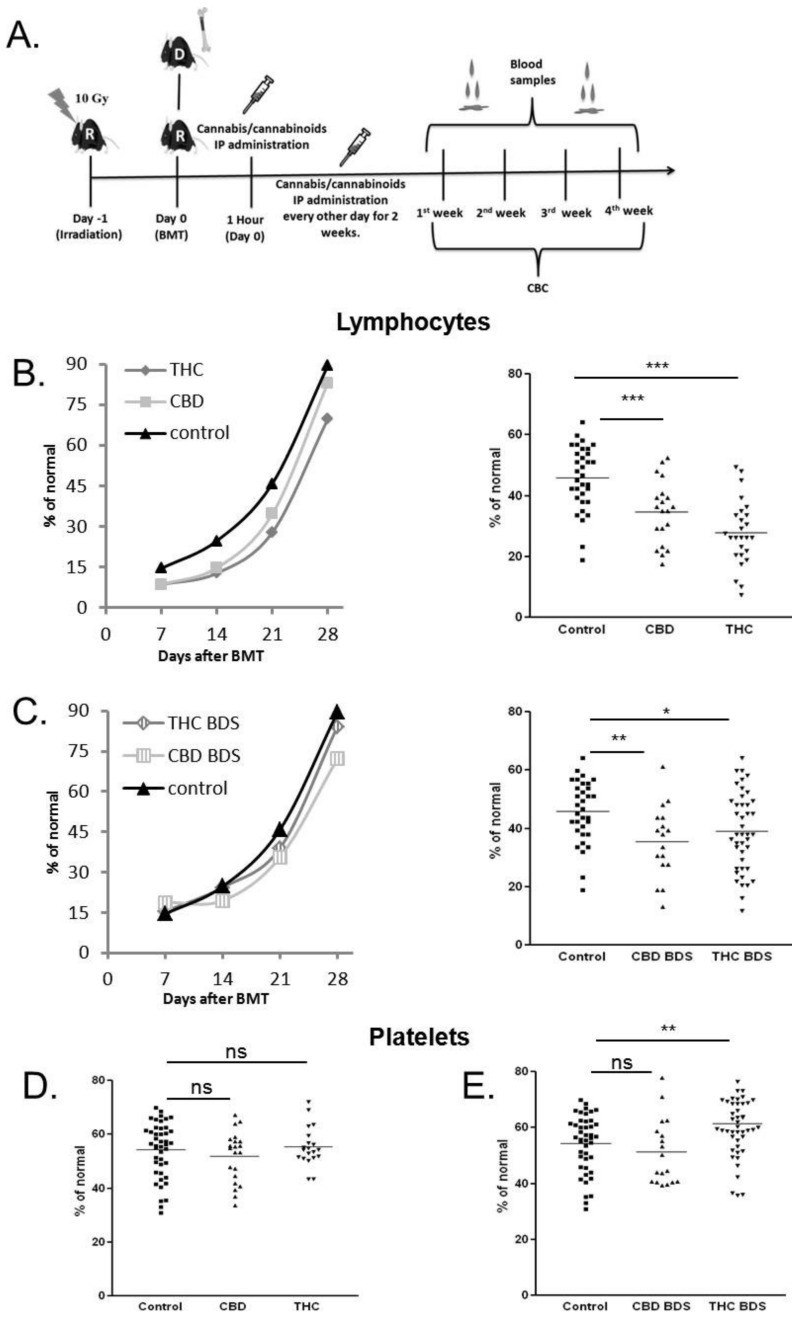
Cannabis/Cannabinoids administration to syngeneic BMT model. (**A**) Recipient C57BL/6 mice received lethal whole-body irradiation and were reconstituted with 8 × 10^6^ donor C57BL/6 bone marrow cells. Cannabis/cannabinoids were administered IP every other day, for 2 weeks from the day of transplantation. Blood samples for CBC were obtained once a week. Average lymphocyte counts in pure cannabinoid-treated groups (**B**) and BDS-treated groups (**C**) are presented. Average counts at different time points (left) and day 21 after transplantation counts (right). (**D**) Average platelet counts in pure cannabinoid-treated groups (left) and BDS-treated groups (right), day 14 after transplantation. Data are summarized from four independent experiments (lymphocytes) and three independent experiments (platelets). *p* Value *, <0.05; **, <0.001; ***, <0.0001. % of normal-% of the mean cell concentrations (cells/µL) in healthy C57BL/6 mice, non act: non-activated, act spl: activated splenocytes, THC: D9 tetrahydrocannabinol, CBD: cannabidiol, BDS: Botanical Drug Substance, ns: not significant.

To investigate the effect of THC, CBD and cannabis extracts on hematopoiesis after BMT, we utilized a syngeneic transplantation model. C57BL/6 mice underwent lethal whole-body irradiation and were reconstituted with 8 × 10^6^ donor C57BL/6 BM cells the following day (Figure 4A). Five mg/kg of cannabis extracts/pure cannabinoids/vehicle were administered intraperitoneally (IP) from the day of transplantation, every other day, for 2 weeks. Once a week, starting 1 week after transplantation, blood was collected from mice tails and CBC with differentials was performed. Both pure cannabinoids and cannabis extracts had a significant inhibitory effect on lymphocyte recovery (Figure 4B,C). Among the tested compounds, pure THC had the strongest effect with a mean of 39% inhibition compared to vehicle-treated mice (control), 3 weeks after transplantation (Figure 4B, right). The inhibitory effect of CBD treatment was significantly lower. Interestingly, there was no significant difference between CBD BDS and THC BDS treatment (Figure 4C, right). The number of monocytes and granulocytes was not affected by the treatment (data not shown). Platelet recovery was significantly improved only in the group that received THC BDS treatment, with a mean of 10% improvement compared to control, 2 weeks after transplantation (Figure 4D,E).

These results demonstrate that cannabis/cannabinoids treatments affect hematological reconstitution after BMT and that different cannabinoid formulations have different effects.

### 2.4. CB2 Receptor Has an Inhibitory Effect on Lymphocyte Recovery

**Figure 5:**
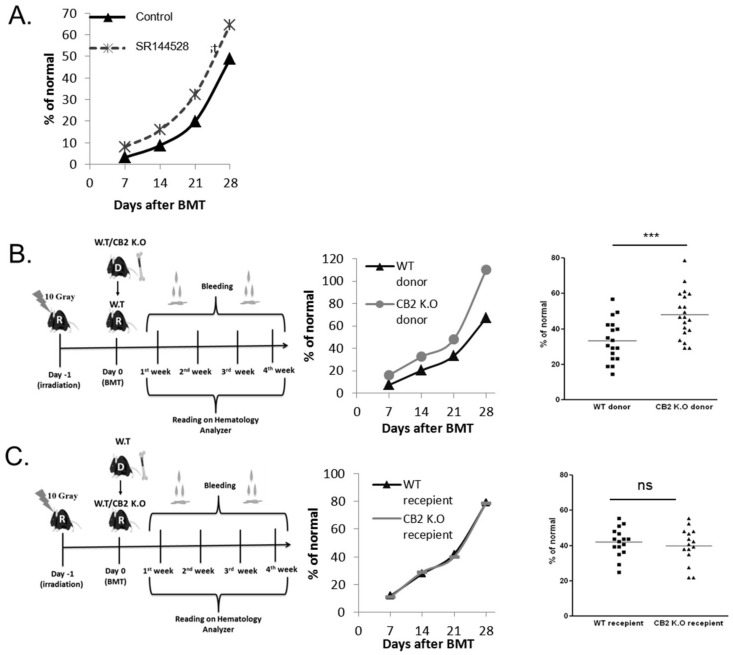
The role of CB2 in lymphocyte recovery. (**A**) Recipient C57BL/6 mice underwent syngeneic BMT. CB2 reverse agonist, SR144528, was administered IP once a day for 1 week from the day of transplantation. Blood samples were obtained once a week. Average lymphocyte counts. (**B**) Syngeneic BMT from CB2 KO donor mice to C57BL/6 WT mice. Average counts at different time points (left) and day 21 after transplantation (right). (**C**) Syngeneic BMT from C57BL/6 WT donor mice to CB2 KO mice. Average counts at different time points (left) and day 21 after transplantation (right). Data are summarized from three independent experiments. *p* Value ***, <0.0001. % of normal-% of the mean cell concentrations (cells/µL) in healthy C57BL/6 mice. WT: wild type, CB2KO: CB2 knock out.

Since THC had the strongest inhibitory effect on lymphocyte recovery, we wanted to examine the involvement of CB2 in this process. First, we administered syngeneic BMT mice with the CB2 inverse agonist SR144528 once a day for 1 week from the day of transplantation. Once a week, starting 1 week after transplantation, blood was collected from mice tails and CBC with differentials was performed. In this model, the mice did not receive any cannabinoid treatment. Our results demonstrate significantly improved lymphocyte recovery in the treated group (Figure 5A).

To clarify whether this improvement is due to an effect on the grafted cells or on the accepting environment, we used CB2 KO mice as donors/acceptors in BMT experiments. The normal blood counts of CB2 KO female mice were similar to the WT C57BL/6 counts (Figure S3B). C57BL/6 mice underwent lethal whole-body irradiation and were reconstituted with 8 × 10^6^ donor CB2 KO or C57BL/6 BM cells the following day. There were a significantly higher number of lymphocytes in the group that received CB2 KO transplant compared to control, starting from the second week after transplantation (Figure 5B). When C57BL/6 BM cells were transplanted to CB2 KO or C57BL/6 recipient mice, lymphocyte counts were not significantly different (Figure 5C).

Altogether, these experiments demonstrate the inhibitory role of CB2 in the recovery of blood lymphocytes after bone marrow transplantation

### 2.5. Cannabis/Cannabinoids Administration for GVHD Prophylaxis

Several studies, as well as our in vitro assays (Figure 1), indicate that cannabinoids have an anti-inflammatory function [8]. Yeshurun, et.al demonstrated the beneficial effect of the cannabinoid CBD in GVHD prophylaxis in patients [16]. We therefore decided to compare the immunosuppressive effect of CBD/THC and cannabis extracts on GVHD prophylaxis in a murine model.

**Figure 6:**
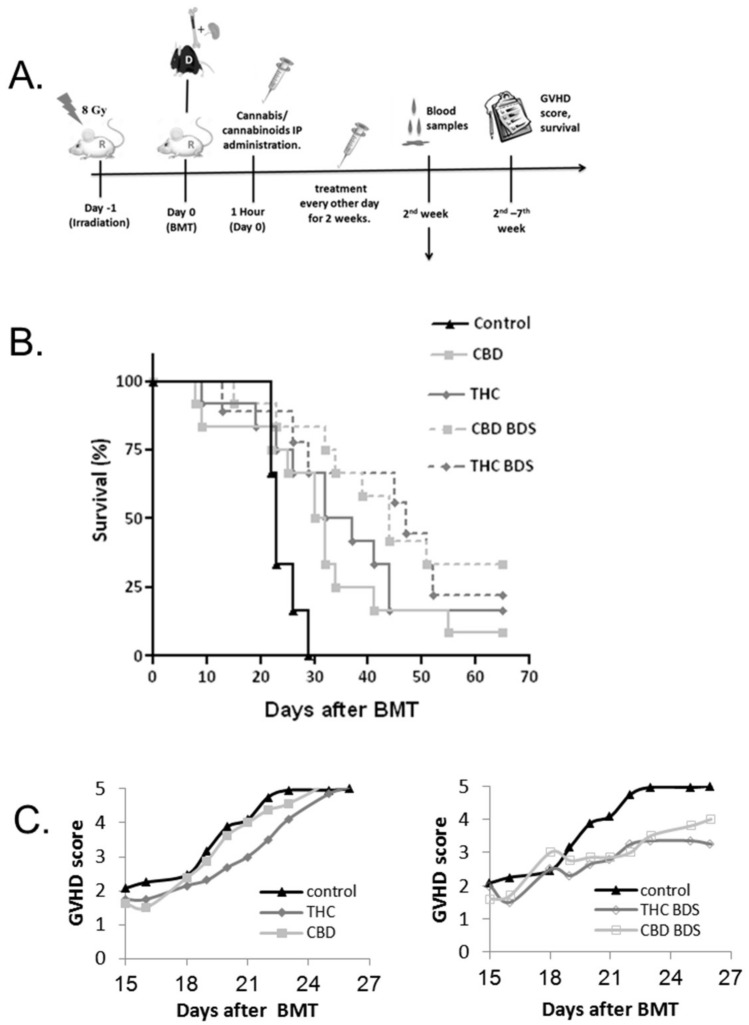
Cannabis/Cannabinoids administration for GVHD prophylaxis. (**A**) Recipient BALB/c mice received lethal whole-body irradiation and were reconstituted with 8 × 10^6^ donor C57BL/6 bone marrow cells and 2 × 10^6^ spleen cells. Cannabis/cannabinoids were administered IP every other day, for 2 weeks from the day of transplantation. The clinical condition of the mice was evaluated for up to 67 days after transplantation. (**B**) Survival curve. Differences between control and THC BDS as well as CBD BDS is significant. The difference between THC and THC BDS is also significant. Data are summarized from two independent experiments, six mice/group in each experiment. (**C**) Average GVHD score (Days 15–26). Differences between THC BDS/CBD BDS to the control group are significant. The same control group is shown in the left and right graph.

BALB/c mice underwent whole-body irradiation followed by allogeneic BMT from C57BL/6 donor mice. Five mg/kg of cannabis extracts/pure cannabinoids/vehicle were administered IP, from the day of transplantation, every other day, for 2 weeks (Figure 6A). Mice chimerism was not affected by the treatment (Figure S5). In our model, both CBD BDS and THC BDS significantly improved survival (Figure 6B, right), while pure cannabinoids had a smaller effect (Figure 6B, left). The difference between THC and THC BDS is significant. Moreover, GVHD scores were significantly lower in mice administered cannabis extracts (Figure 6C).

These results demonstrate that cannabis extracts are more potent modulators of allogeneic activation in vivo than pure THC or CBD.

## 3. Discussion

Cannabis contains hundreds of chemical compounds [2]. Different sub-strains of cannabis comprise unique sets of cannabinoids and other molecules which influence the clinical outcome of the treatment. The scientific data regarding the use of a specific strain or isolated cannabinoid for the treatment of each disease is currently very limited.

The increased demand for medical cannabis around the world results in an urgent need for scientific evaluation of cannabinoids-based medicines as a therapy. In this study, we decided to compare the effect of the most abundant cannabinoids, THC and CBD, and to also examine cannabis extracts from THC- and CBD-rich plants. We used the extracts because they are most commonly used by patients and also because of the suggested entourage effect [17]. We have used in vitro assays as well as syngeneic and allogeneic murine models to test the effect of these cannabis-based treatments on BMT. Our results demonstrate that all of these cannabinoid-based treatments suppress lymphocyte proliferation and influence cytokine secretion. Decreased surface expression of the early activation marker CD69 was evident in higher concentrations of treatment. CBD, CBD BDS and THC BDS significantly inhibited the elevation of %CD8 in the culture upon activation. In accordance with its known anti-inflammatory activity [5], CBD had the most profound effect on cell proliferation. The induction of IL-10 together with inhibition of IL-17 secretion by all treatments may indicate an influence on the Th17/Treg balance. Th17 cells are known to participate in the pathophysiology of GVHD [24] and several autoimmune diseases and, therefore, this effect is most clinically relevant. Notably, our results resemble previous data for cannabinoid treatment in an experimental autoimmune encephalomyelitis (EAE) mice model for multiple sclerosis and in an animal model of asthma [25,26]. IL-10 elevation and IL-17 reduction were also evident in cultured CD4^+^ T cells from cannabinoid addicts [27]. Interestingly, we did not find any correlation between the effect of the treatment on cytokine secretion and its effect on proliferation. For example, 10 µg/mL THC BDS reduced cell proliferation by only 25%, but induced a relatively high secretion of the regulatory cytokine IL-10.

CD69 surface expression, 4h after stimulation, was only slightly affected by the cannabinoid-based treatments in 3–5 µg/mL concentrations. Although CD69 is a well-known activation marker, it was also found to be involved in downregulation of the immune response through controlling the production of the pleiotropic cytokine transforming growth factor-β (TGF-β) [28], which has a role both in Treg and in Th17 differentiation. Therefore, these results correspond with our results of the late activation markers tested, i.e., proliferation and cytokine secretion.

We utilized CB2 knockout mice and antagonists/inverse agonists of different receptors to screen for signal transduction mediators used by CBD and THC to inhibit lymphocyte activation. We found that CB2 was the main mediator of THC’s effect but was not involved in the effect of CBD. The tested TRP channels we examined and GPR55 were also not found to mediate CBD’s inhibitory function. PPARγ was found to mediate part of CBD’s inhibitory effect on lymphocyte activation. PPARγ is a nuclear hormone receptor widely expressed in adipose tissue and in immune/inflammatory cells, colonic mucosa and placenta [29]. PPARγ activation attenuates inflammatory processes associated with several diseases and it was found to be involved in the inhibition of Th17 differentiation [30]. The involvement of PPARγ in CBD signaling has been shown in various tissues [21]. For example, in biopsies from patients with ulcerative colitis, CBD treatment ex vivo reduces signs of inflammation that can be blocked with a PPARγ antagonist [31]. We also found that CBD is able to activate another nuclear receptor, AhR. This activation may contribute to CBD’s effect on T cell differentiation. The involvement of other receptors in CBD-related signaling in lymphocytes has yet to be found.

There are several obstacles to a good clinical outcome of BMT. The toxicity of the conditioning protocol leads to a period of low hematological counts which makes the patients susceptible to common and unusual infections [14]. Our results demonstrate that all the cannabinoids-based treatments we have used significantly delay lymphocyte reconstitution after transplantation. This finding is of great importance since delayed lymphocyte re-constitution may have a deleterious effect on the clinical outcome. On the other hand, THC BDS treatment improved platelet recovery. The involvement of endocannabinoids in thrombogenesis was previously demonstrated [32,33,34]. However, it is not known yet which component is responsible for this effect in our model and if this result can be repeated with THC BDS from different sources.

The finding that cannabinoids-based treatments inhibited lymphocyte recovery rather than provoked it was unexpected. Patinkin et al. demonstrated that endocannabinoids increase the number of several hematopoietic cell’s colony-forming units (CFU) in vitro [32], and Jiang et al. showed elevation of CFU in bone marrow of sub-lethally irradiated mice treated with the CB2 agonist AM1241 [35]. Importantly, our results clearly identify CB2 as an inhibitory receptor of lymphocyte recovery. We demonstrate that THC, a CB2 agonist, has the strongest inhibitory effect on lymphocyte recovery. CB2 antagonist treatment in syngeneic transplanted mice improved lymphocyte recovery, and similarly, CNR^−/−^ bone marrow transplanted into WT mice resulted in improved recovery of lymphocytes. Wild type bone marrow transplanted into CNR^−/−^ mice did not affect the recovery rate, indicating a role for CB2 expression on the transplanted cells rather than on the cells of the accepting environment. Our results can possibly be explained by the role of cannabinoids in hematopoietic stems and progenitor cell homing to the bone marrow niche. Kose et al. recently demonstrated that endocannabinoids can stimulate the migration of human hematopoietic stem cells in a cannabinoid receptors-dependent manner [36]. They also showed that the concentration of the endocannabinoid 2AG in blood plasma is higher than in bone marrow plasma, in healthy individuals. Pereira et al. proved that CB2 has a role in the retention of immature B cells in bone marrow [37], and Hoggatt et al. demonstrated a significant decrease in CXCR4 in bone marrow cells treated with the CB1/CB2 agonist CP55940 [10]. Together, these results point to an important role for the endocannabinoid system in the migration of the hematopoietic stem and progenitor cells, an issue that requires further investigation.

In contrast to the greater effect of the pure cannabinoids in vitro and in the syngeneic transplantation model, the cannabis extracts had more of an effect on GVHD prophylaxis. This result together with the cytokine results from our in vitro experiments and the syngeneic model experiments demonstrate that the effects of the extracts are different from the effects of pure cannabinoids. There are two possible explanations for this phenomenon. The unique effects of the extract could result either from other molecules in the plant (not THC/CBD) or from a synergistic function of THC/CBD with other molecules.

The clinical use of medical cannabis and cannabinoids is different from other evolving medications because it is administered to patients despite the shortage of scientific pre-clinical research-based evidences. Our results highlight the complexity of using cannabinoids-based drugs and the need for additional comparative scientific results. The results of this study may influence the treatment of BMT patients with cannabinoids-based medicines by facilitating the choice of which particular drug should be used to treat their specific clinical condition.

## 4. Materials and Methods

### 4.1. Cannabis Extracts and Cannabinoids

This research was performed under the approval of The Medical Cannabis Unit in the Israeli Ministry of Health (REQ46). Pure THC was kindly provided by the laboratory of Prof. Raphael Mechoulam. Synthetic CBD was purchased from STI Pharmaceuticals LTD., Newtown, UK. Cannabis Sativa and Indika extract with high (20–30%) content in THC or CBD (i.e., THC-BDS/CBD BDS respectively) were supplied by Cannabliss (Cannabliss LTD., Tel Aviv, Israel). This company is authorized by the Israeli ministry of health to supply medical cannabis products to patients and for research. Extraction was obtained using Ethanol, and evaporated. The THC, CBN and CBD contents of the extract were quantified against a commercial THC, CBN and CBD standards (Izun Pharma, Jerusalem, Israel). THC BDS: 20–30% THC, 1% > CBD, 1% > CBG, 2% > CBN. CBD BDS: 1% > THC, 20–30% CBD, 1% > CBG, 2% > CBN.

### 4.2. Inhibitors

SR144528—a CB2 receptor antagonist, was purchased from Abcam, Cambridge, UK. A967079—a TRPA1 Receptor antagonist and BCTC—a TRPV1 Receptor antagonist, were purchased from Alomone Labs, Jerusalem, Israel. GSK2193874—a TRPV4 antagonist, was purchased from SIGMA-ALDRICH, Rehovot, Israel. CID16020046—a GPR55 antagonist, was purchased from Cayman, MI, USA and GW9662—a PPARγ antagonist, was purchased from Enzo Life Sciences, New York, NY, USA.

### 4.3. Mice

Female 8- to 11-week-old C57BL/6 and BALB/c mice were purchased from Envigo, Jerusalem, Israel, and Cannabinoid Receptor 2 (CB2) knockout mice (CNR2^−/−^) [38] were bred in the specific pathogen-free (SPF) facility of the Authority of Biological and Biomedical Models at the Hebrew University of Jerusalem. The study was approved by the Institutional Animal Care and Use Committee of the Hebrew University of Jerusalem in accordance with national laws and regulations for the protection of animals (MD-15-14619-5, approved 16 July 2016), and the mice were housed under specific SPF conditions.

### 4.4. Lymphocyte Activation Assays

CFSE assays-spleens were harvested from healthy C57BL/6 or BALB/c mice. Splenocytes were centrifuged on a Ficoll-Paque gradient (Fresenius Kabi Norge AS, Oslo, Norway); mononuclear cells were isolated from the interphase layer washed and labeled with carboxyfluorescin diacetate succinimidyl ester (CFSE). A total of 1 × 10^6^ labeled cells/well were plated in 96-well flat bottom plates with RPMI 1640 medium supplemented with 10% FCS, 1% penicillin/streptomycin, and 1% l-glutamine (Biological Industries, Beit Haemek, Israel). Splenocytes were activated with 1 µg/mL anti-CD3 antibodies (Biolegend, San Diego, CA, USA) in the presence of the indicated concentrations of cannabis extracts/cannabinoids for 4 days. CFSE levels in the cells were determined using FACS analysis. The CD8/CD4 ratio was determined by anti-CD4 and anti-CD8 florescent antibodies staining (Biolegend, San Diego, CA, USA) followed by FACS analysis. Cytokine concentration in the culture media was quantified using ELISA Ready SET Go kits (eBioscience, San Diego, CA, USA), according to the manufacturer’s instructions. All determinations were made in triplicates.

CD69 expression assays-spleens were harvested from healthy C57BL/6 mice. A total of 1 × 10^6^ labeled cells/well were plated in 96-well flat bottom plates with RPMI 1640 medium supplemented with 10% FCS, 1% penicillin/streptomycin, and 1% l-glutamine (Biological Industries). Splenocytes were activated with 1 µg/mL anti-CD3 antibodies (Biolegend) in the presence of the indicated concentrations of cannabis extracts/cannabinoids for 4 days. CD69 surface expression was determined by anti-CD69 florescent antibodies staining (Biolegend) followed by FACS analysis.

### 4.5. RNA Extraction and Real Time PCR Analysis

Total cellular RNA was extracted using RNeasy^®^ Mini Kit columns (QIAGEN, Hilden, Germany) according to the manufacturer’s protocols. One microgram of total RNA was used to synthesize cDNA using the High-Capacity cDNA kit (Applied Biosystems, Foster city, CA, USA). The detection of transcript levels of cyp1a1 were performed using the TaqMan Gene Expression Assay Kit (Applied Biosystems), using hprt1 as a reference. All primers were purchased from Applied Biosystems. Real-Time PCR reactions were conducted using StepOne Plus (Applied Biosystems). Data was analyzed by StepOne Software version 2.2 (Applied Biosystems).

### 4.6. Syngeneic BMT Model

C57BL/6 or CB2 knockout mice underwent lethal whole-body irradiation by single exposure to 10 Gy and were reconstituted with 8 × 10^6^ donor C57BL/6 or CB2 knockout BM cells the following day. Cannabis extracts/cannabinoids (5 mg/kg) were administered intraperitoneally (IP), every other day from the day of transplantation, for 2 weeks. Once a week, blood was collected from the mice tail into Ethylenediaminetetraacetic acid (EDTA)-coated capillary tubes. A complete blood count (CBC) with differentials was performed using a validated BC-2800Vet Auto Hematology Analyzer (Mindray, Shenzhen, China).

### 4.7. Allogeneic BMT Model

Balb/c mice underwent lethal whole-body irradiation by single exposure to 8 Gy and were reconstituted with 8 × 10^6^ donor C57BL/6 BM cells and 2 × 10^6^ spleen cells the following day. Cannabis extracts/cannabinoids (5 mg/kg) were administered IP every other day from the day of transplantation, for 2 weeks. For GVHD evaluation, mice were monitored daily for weight loss, diarrhea, ruffled skin, and survival. GVHD score, based on all of the aforementioned factors (rated on a scale of 0–4), was calculated as previously described [39].

### 4.8. Statistical Analysis

Data from the BMT studies are described as mean values on the dot plot, showing individual values (lymphocyte and platelet count) at the indicated time point. Linear graphs show mean values of the same groups of mice at different time points. Data from in vitro studies are represented as mean ± SE. The mean was calculated from the indicated number of experiments. The mean of triplicates from each experiment was used for this calculation. Single comparisons to control were made using two-tailed Student’s *t*-test, with *p* value < 0.05 considered statistically significant. In vitro proliferation tests, CD4, CD8 tests and the survival of the mice in the different treatment groups in the GVHD assay were compared using one tailed ANOVA test + Bonferroni’s multiple comparison, with a *p* value < 0.05 considered statistically significant.

## Supplementary Materials

Supplementary materials can be found at http://www.mdpi.com/1422-0067/20/3/668/s1.
