# Supplementary figures and images for: Cannabinoids Reduce Inflammation but Inhibit Lymphocyte Recovery in Murine Models of Bone Marrow Transplantation
Source: Int J Mol Sci. 2019 Feb 4;20(3):668. doi: 10.3390/ijms20030668 (PMC6387311; doi:10.3390/ijms20030668)

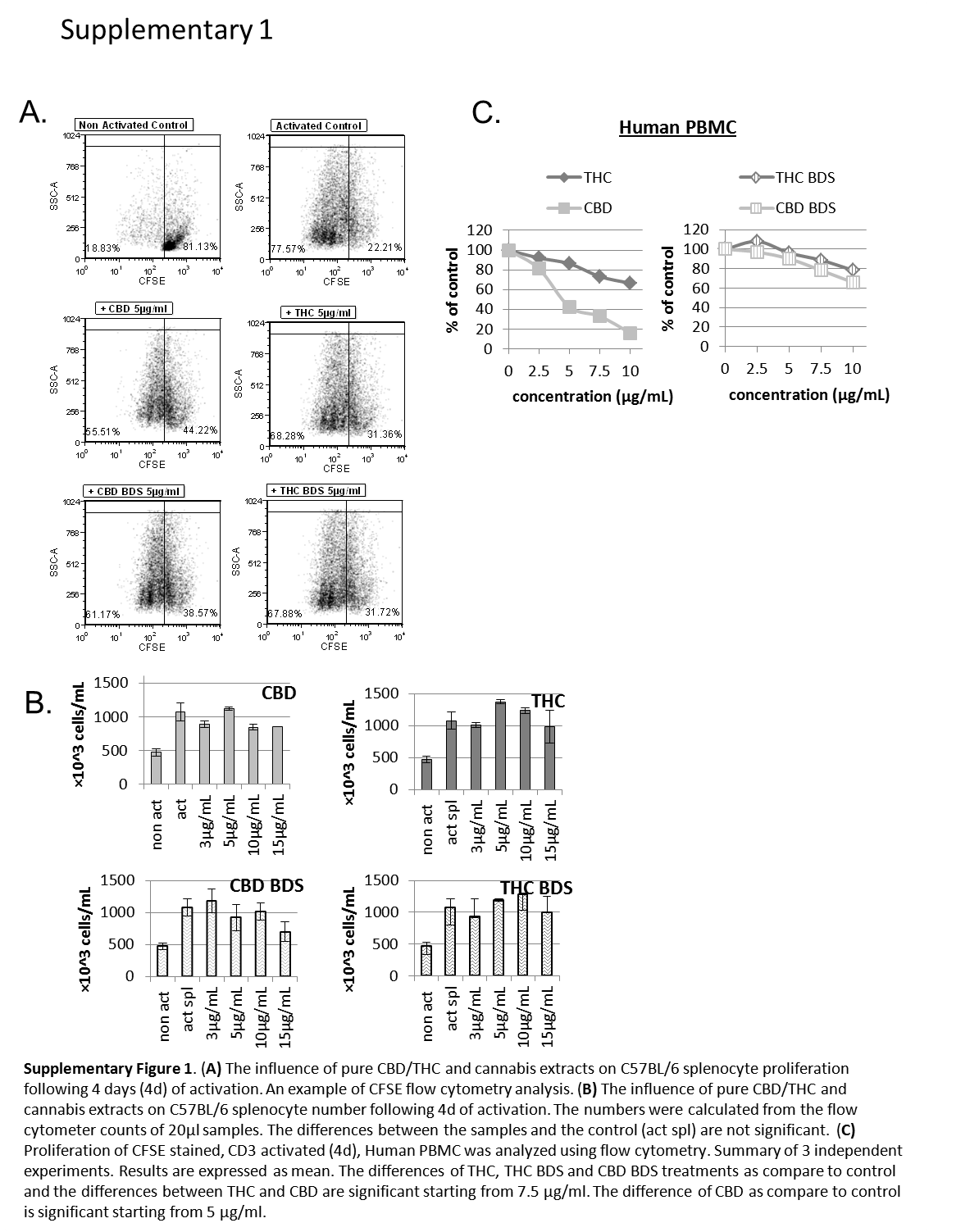

Supplement: Supplementary file 1 [file ijms-20-00668-s001.zip › S1.png]

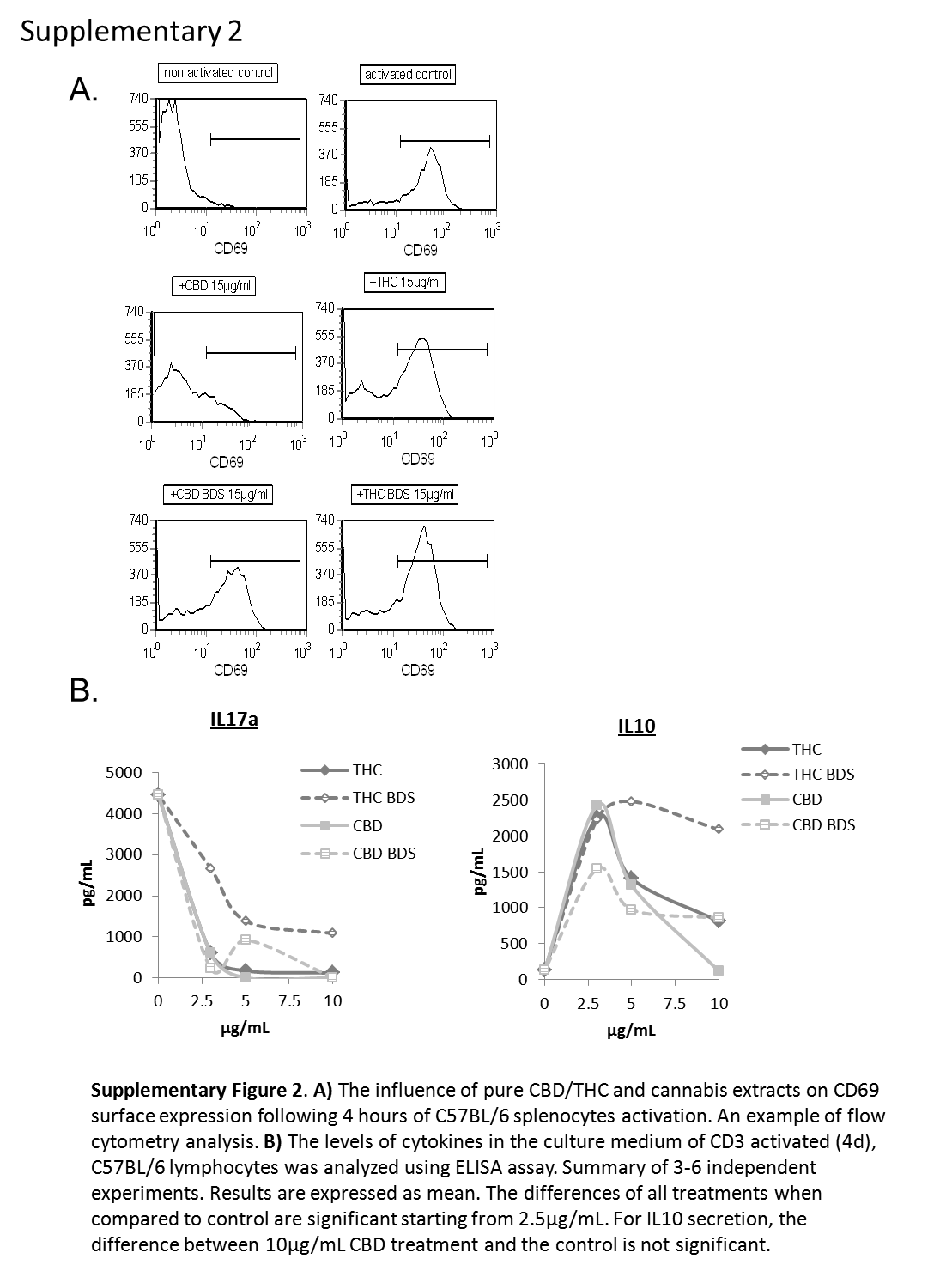

Supplement: Supplementary file 1 [file ijms-20-00668-s001.zip › S2.png]

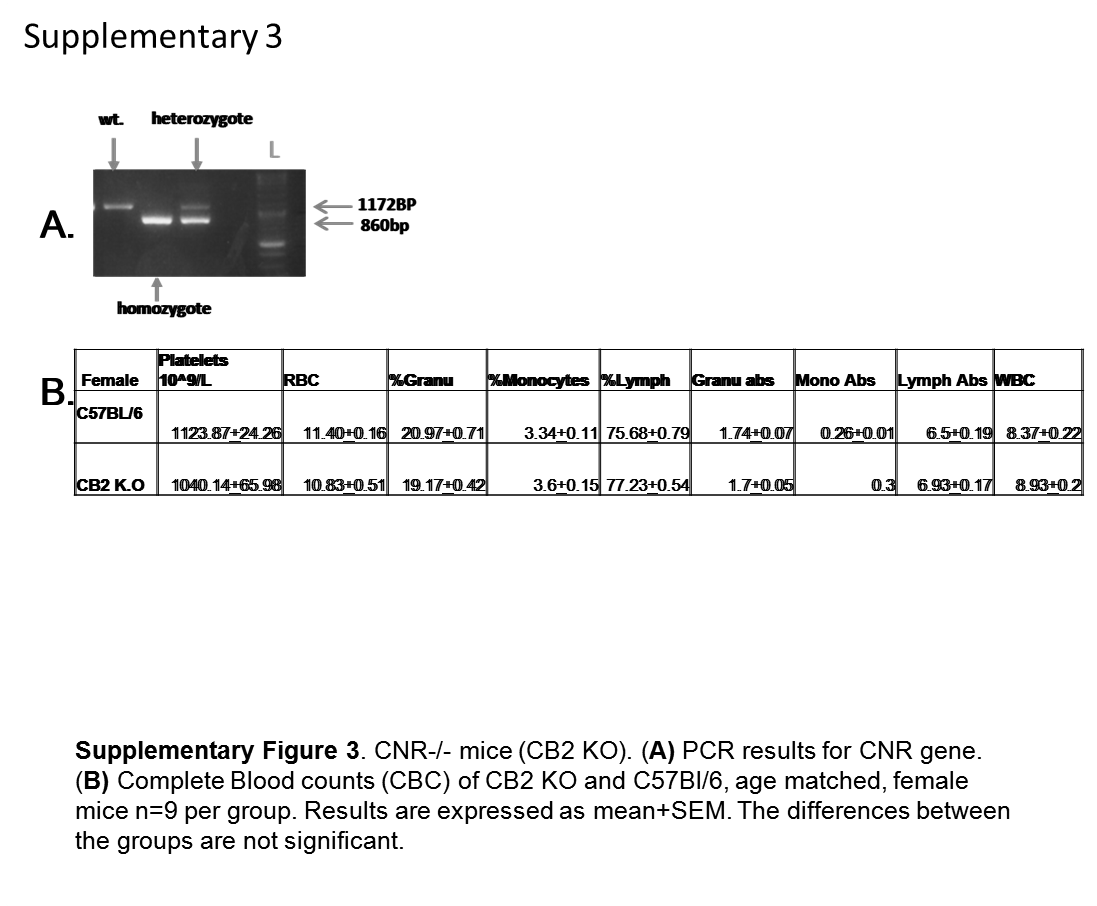

Supplement: Supplementary file 1 [file ijms-20-00668-s001.zip › S3.png]

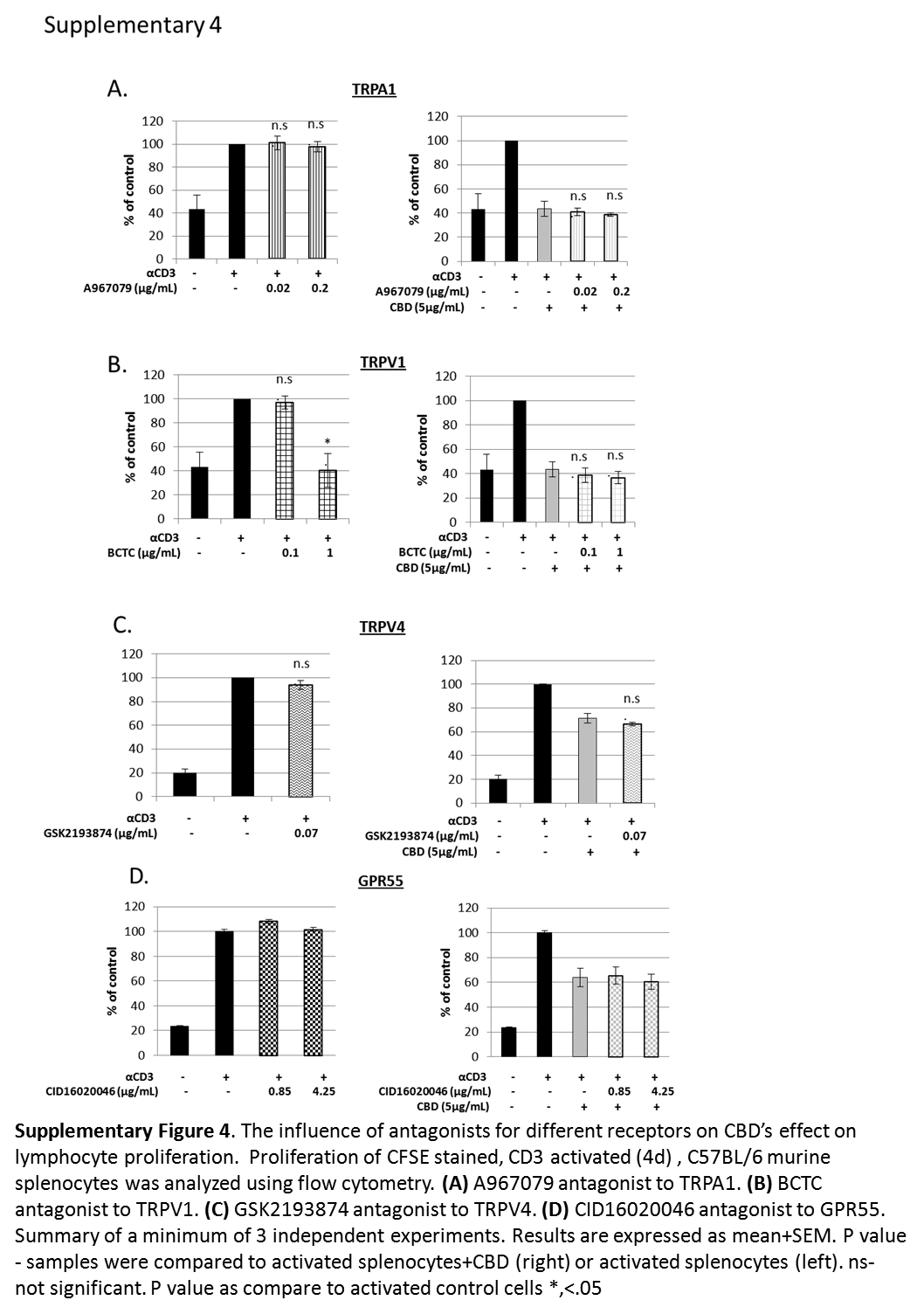

Supplement: Supplementary file 1 [file ijms-20-00668-s001.zip › S4.png]

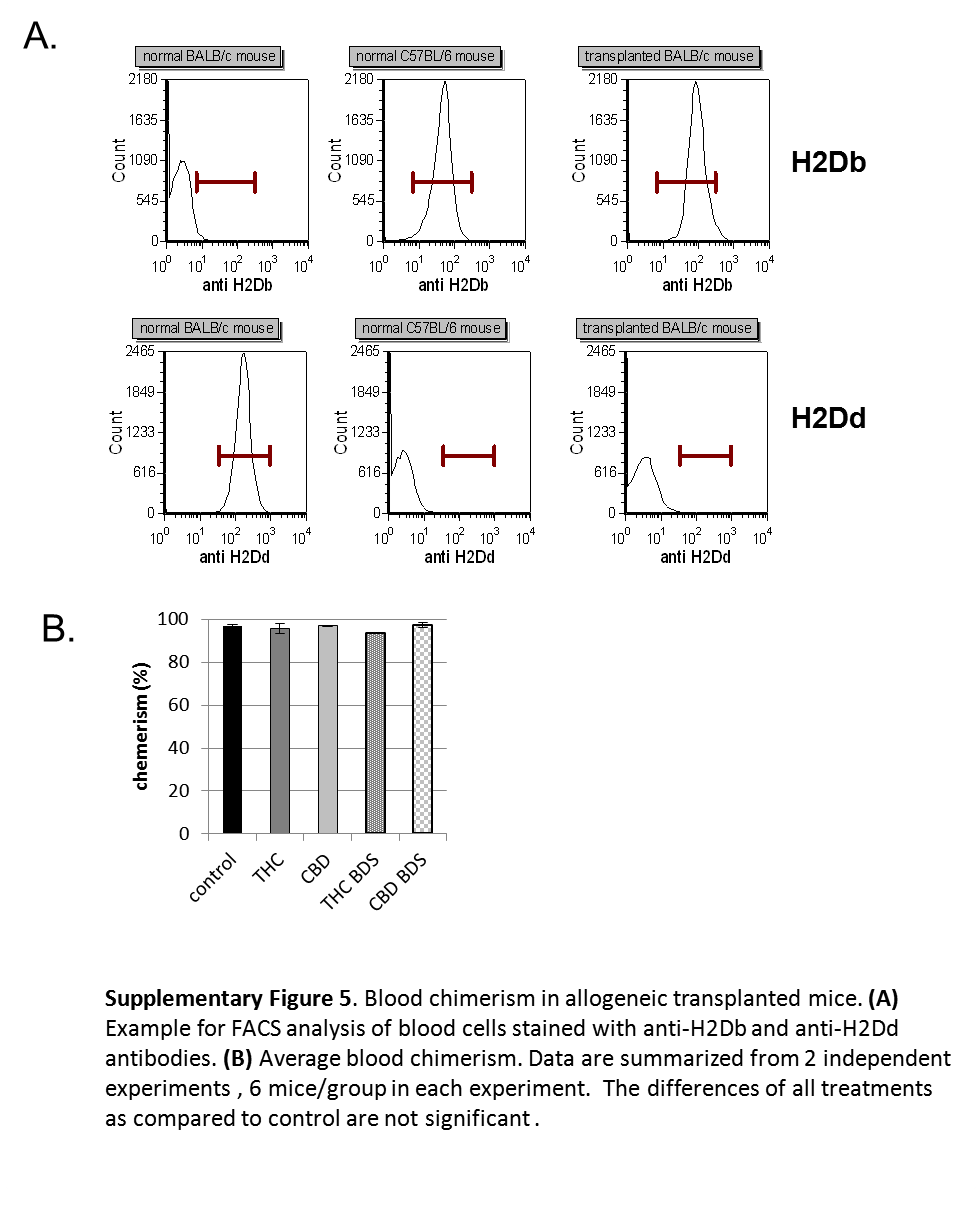

Supplement: Supplementary file 1 [file ijms-20-00668-s001.zip › S5.png]
